# The Role of Abdominal Drain Cultures in Managing Abdominal Infections

**DOI:** 10.3390/antibiotics11050697

**Published:** 2022-05-20

**Authors:** Jan J. De Waele, Jerina Boelens, Dirk Van De Putte, Diana Huis In ‘t Veld, Tom Coenye

**Affiliations:** 1 Department of Intensive Care Medicine, Ghent University Hospital, 9000 Ghent, Belgium; 2 Department of Internal Medicine and Pediatrics, Faculty of Medicine and Health Sciences, Ghent University, 9000 Ghent, Belgium; 3 Department of Medical Microbiology, Ghent University Hospital, 9000 Ghent, Belgium; jerina.boelens@ugent.be; 4 Department of Diagnostic Sciences, Ghent University, 9000 Ghent, Belgium; 5 Department of Gastrointestinal Surgery, Ghent University Hospital, 9000 Ghent, Belgium; dirk.vandeputte@ugent.be; 6 Department of Internal Medicine and Infectious Diseases, Ghent University Hospital, 9000 Ghent, Belgium; diana.huisintveld@ugent.be; 7 Laboratory of Pharmaceutical Microbiology, Ghent University, 9000 Ghent, Belgium; tom.coenye@ugent.be

**Keywords:** antibiotics, antimicrobials, antimicrobial stewardship, infection, drain

## Abstract

Intra-abdominal infections (IAI) are common in hospitalized patients, both in and outside of the intensive care unit. Management principles include antimicrobial therapy and source control. Typically, these infections are polymicrobial, and intra-operative samples will guide the targeted antimicrobial therapy. Although the use of prophylactic abdominal drains in patients undergoing abdominal surgery is decreasing, the use of drains to treat IAI, both in surgical and non-surgical strategies for abdominal infection, is increasing. In this context, samples from abdominal drains are often used to assist in antimicrobial decision making. In this narrative review, we provide an overview of the current role of abdominal drains in surgery, discuss the importance of biofilm formation in abdominal drains and the mechanisms involved, and review the clinical data on the use of sampling these drains for diagnostic purposes. We conclude that biofilm formation and the colonization of abdominal drains is common, which precludes the use of abdominal fluid to reliably diagnose IAI and identify the pathogens involved. We recommend limiting the use of drains and, when present, avoiding routine microbiological sampling.

## 1. Introduction

Abdominal infections are the second most frequent type of infection in intensive care units (ICU) and are typically caused by the perforation of the gastrointestinal (GI) tract. The outcome is dependent on the severity of illness and risk factors for mortality include older age, liver and heart failure, and antimicrobial resistance, among other factors [1]. Apart from organ support, when necessary, antimicrobial treatment and source control are the cornerstones of therapy for abdominal infection [2].

The microbiology of intra-abdominal infections is typically polymicrobial, with a wide range of pathogens involved, including anaerobic and aerobic Gram-positive and Gram-negative bacteria [3]. As the presence of multidrug-resistant (MDR) pathogens is unpredictable, intra-operative/intra-procedural cultures are important to ensure the adequate coverage of the antimicrobial therapy, as well as timely antimicrobial de-escalation [4].

In patients with abdominal infection requiring surgery, as well as in patients undergoing elective surgery, abdominal drains are often used to ensure the adequate drainage of postoperative fluid collections or residual purulent effusion. Also for other indications in the absence of infection, e.g., ascites in patients with chronic liver disease or malignancy, abdominal drains may be inserted percutaneously. Frequently, particularly in patients in ICU and/or with protracted courses, a microbiological analysis of samples of abdominal drains or fluid collections is performed. While it may be tempting to obtain samples from these sites, experience dictates that while cultures of these samples often come back positive, equally often it is unclear whether this is truly a sign of a clinical infection and whether the microorganisms found are representative of the cause of infection.

In this narrative review, we will discuss the role of abdominal drains in surgery, both in the presence and absence of infection, highlight important considerations regarding biofilm formation, review the clinical evidence available, and provide practical guidance for the interpretation of drain culture results as well as the integration of this in the overall management of the patient.

## 2. The Role of Drains in Abdominal Surgery

### 2.1. Prophylactic Use

Controversy has existed for years and persists to this day around the use of prophylactic drains in abdominal surgery [5,6]. The beliefs that draining the surgical site to avoid fluid accumulation is essential or that the drain may give sentinel information about complications (e.g., bleeding or GI tract leakage) lack evidence. Moreover, drains are susceptible to retrograde contamination and can cause pain as well as ascites formation due to irritation. This is reflected in ‘Enhanced recovery after surgery’ (ERAS) protocols, where the routine use of drains is not recommended [7]. In addition, surgical drain dislocation is frequently encountered in patients undergoing digestive abdominal surgery [8]. Drains, of course, are never a substitute for meticulous surgery [9].

The indication for prophylactic drainage of the abdominal cavity has been evaluated in several types of abdominal surgery. There is no consensus about routine usage of drains in pancreatic and rectal surgery. A meta-analysis conducted by Huttner et al. concluded that pancreatic resection, with or without abdominal drainage, results in similar rates of mortality, morbidity, and reintervention [10]. Despite the growing evidence against the routine use of drains in surgery, there is a subgroup of patients for whom drains may play a beneficial role [11,12]. For pancreatic surgery, a systematic review concluded that the most conservative approach is the routine placement of a drain and early removal thereof, unless the patient’s clinical course or drain fluid amylase concentration suggests the development of a fistula [13]. For rectal surgery, some studies suggest that the routine use of pelvic drains does not confer any significant advantage in terms of postoperative complications [14,15], while a recent systematic review concluded that the use of a drain is warranted [16]. The ‘Ileus Management International Study’ found that more than one-third of participating centers across the world routinely used drains in most colorectal resections [17].

In the literature, there is evidence that the routine use of a drain after minimally invasive right hemicolectomy [18], bariatric surgery [19,20], splenectomy [21], or laparoscopic cholecystectomy for non-complicated benign gallbladder disease [22] does not reduce postoperative morbidity. Routine prophylactic drains in laparoscopic total gastrectomy are not necessary in most patients, although a prophylactic drain may be useful in high-risk patients [23]. The routine use of abdominal drainage to reduce postoperative complications after appendectomy for complicated appendicitis remains controversial, as shown in a recent meta-analysis [24].

Some authors conclude that drains need only be used in emergency situations [6], but that, even in duodenal perforation, the advantage of using a drain has not been demonstrated [25].

### 2.2. Therapeutic Use

The therapeutic use of abdominal drains is an entirely different issue. In the treatment of abdominal infections, abdominal drains are indicated to drain residual infected fluid after emergency abdominal surgery, e.g., in case of generalized (fecal) peritonitis, perforated diverticulitis [26], anastomotic leakage [27], complicated Crohn’s disease [28], delayed posttraumatic perforation, or ischemic perforation. Source control encompasses all measures to eliminate the infection and, as such, surgical or non-surgical drainage is often part of the treatment [29]. In abdominal sepsis, the absence of source control, as well as delayed or inadequate source control, is an independent predictor of poor outcomes [30].

However, not all abdominal infections require operative surgical interventions. The preferred way to drain an abdominal abscess is percutaneous (often ultrasound or CT guided) drainage [31], such as in the treatment of complicated diverticulitis, where drainage may be the only intervention required. In addition, endoscopic ultrasound guided drainage can be considered in selective cases [32]. In these settings, the drain is one of the cornerstones of the therapy, alongside antimicrobial therapy.

The drainage of abdominal abscesses may not always be necessary; for example, small postoperative abscesses (less than 2 cm^3^) can be managed without intervention or drain after appendectomy [33]. In addition, in diverticular disease, small abscesses can be treated with antibiotics, but larger abscesses of 3–5 cm in diameter should be drained percutaneously [34].

## 3. Understanding the Importance of Biofilm Formation in Abdominal Drains

Historically, microbial cells have been considered as free-living (planktonic) and not interacting much with their environment and other cells, but extensive research over recent decades has shown that this is not the case. In vivo microorganisms will typically occur as biofilms, communities of microorganisms embedded in a self-produced matrix that can occur attached to a surface or as aggregates embedded in host tissue [35,36]. Microorganisms embedded in biofilms show a drastically reduced susceptibility to antimicrobial agents (antibiotics and disinfectants), which is not only due to their well-known resistance mechanisms (including efflux, the enzymatic inactivation of the antibiotic and target modification), but is also related to the biofilm-specific tolerance mechanisms (including metabolic changes and reduced transport of antimicrobials into the biofilm) [37,38]. In addition, biofilm-associated microorganisms are not easily cleared by the immune system [39]. Biofilm formation on (implanted) medical devices is a well-known problem that has a substantial impact on morbidity and mortality [40]. The risk factors for the development of such chronic biofilm-related infections associated with implanted devices include immunomodulatory therapy (steroids), diabetes, smoking, and renal disease (hemodialysis); these risk factors suggest that overall, a compromised innate immune response increases the risk for such infections [41].

Biofilm formation on medical devices presents physicians and microbiologists with two main problems: i.e., (i) how can we diagnose such an infection and (ii) what does it mean for the treatment of the patient? The answers to these questions largely depend on the type of medical device and the site of infection and specific recommendations for abdominal drains are not available. However, general guidelines for the diagnosis and treatment of biofilm-related infection are available [42] and can help guide the decision-making process in the case of biofilm-related infections associated with the use of abdominal drains.

For diagnosing a device-related biofilm infection, accurate sampling is a prerequisite. In the case of catheter-related infections, the catheter tip can be cultured using quantitative or semi-quantitative methods. In the quantitative approach, it is recommended that samples are vortexed and sonicated prior to culture, while the semi-quantitative approach requires rolling the catheter tip on an agar plate. For devices that cannot easily be removed (e.g., central venous catheter), the sampling of debris (if present/visible), swabbing of the internal surface, and/or injection of sterile saline followed by recovery and culture of the fluid are recommended [42]. There is no consensus on what the best approach is, although a combination of approaches (including internal surface cultures) has been suggested to give the best results [43]. Whether or not it is useful to sample and culture abdominal fluid through an abdominal drain is unclear, but if this is carried out, it seems reasonable to do it with freshly recovered liquid (in line with the recommendation to sample freshly obtained urine in case of biofilm urinary tract infections in patients with indwelling urinary catheters). However, several studies have indicated that an analysis of urine leads to a considerable underestimation of biofilm formation on urinary catheters [44,45], so the value of sampling ascites to assess biofilm formation on the surface of the drain remains to be determined.

A second important issue regards how the observation of a biofilm in the context of an abdominal drain impacts the treatment of the patient. It is well established that results from conventional antimicrobial susceptibility testing (i.e., determination of the MIC or measurements of zone diameters) are a poor predictor of antimicrobial activity against biofilms, due to biofilm-specific tolerance mechanisms [37,38], as well as the pronounced differences between the physicochemical conditions encountered by the bacteria in vitro and in vivo [46,47]. In addition, several studies have shown that measuring alternative parameters of antimicrobial activity in vitro (e.g., determining minimum biofilm inhibitory concentration instead of MIC) does not always lead to a better prediction of activity in vivo and/or a better clinical outcome [48,49] and there is currently no evidence that using standardized biofilm susceptibility testing in clinical microbiology laboratories would improve patient outcomes [42,47].

Finally, it is worth mentioning that considerable effort is devoted to developing materials for medical devices that are refractory to bacterial colonization and biofilm formation, by the inherent modification of the surface (e.g., making it more hydrophobic) and/or by developing materials that release antimicrobial agents such as silver, chlorhexidine, or antibiotics [50,51]. However, very few studies have been conducted with surface-modified abdominal drains; in one such study, the phospholipid impregnation of rubber drains reduced the adherence of *Escherichia coli* and *Enterobacter cloacae* in an in vitro set-up [52]. In a more recent clinical study, standard drains were compared to drains covered with a biodegradable polyesteramide (coladerm) or chlorhexidine [53]. Drains were removed 2–14 days after implantation and bacteria were recovered from 66% (standard), 30% (coladerm covered) and 12% (chlorhexidine) of the drains, suggesting that antimicrobial abdominal drains might reduce the incidence of biofilm formation.

## 4. Clinical Data

While the abdominal drain fluid is frequently sent for culture, the clinical data are limited in helping us to understand how the results should be interpreted. In contrast to other aseptically inserted drainage systems, postoperative abdominal drains are often placed in highly contaminated areas, e.g., in patients with peritonitis after GI tract perforation or postoperative abdominal abscesses. Therefore, we can assume that the microorganisms that were present at the surgical site at the moment of drain placement will rapidly colonize the surface of the drain and any collection devices [54]. Furthermore, the risk of ascending infections exists in the days following drain insertion [55,56] and this may even be exacerbated by flushing or rinsing the catheter.

De Ruiter et al. cultured abdominal drains from patients with different sources of infection on a weekly basis and found that the culture was often positive, with Gram-positive microorganisms encountered increasingly more frequently after 4 weeks, while Gram-negative bacteria decreased [57]. Perez et al. reported high rates of positive cultures in liver transplant patients; the drain tip culture was positive in 83% of the patients, in whom coagulase-negative staphylococci were most frequently isolated (31%). They could not link colonization to subsequent infection [58].

Chisena et al. identified *Staphylococcus epidermidis* as the most important colonizer of indwelling abdominal catheters and a subsequent cause of infection [59], but this observation was contested by Prieto-Borja et al. The latter group sonicated and cultured intra-abdominal drains from patients after routine laparotomy and laparoscopic surgery without postoperative intra-abdominal sepsis [56]. They concluded that only the presence of non-skin microbiota was associated with infection, complication, and a worse outcome for the patient. They also observed a correlation between the duration of the drainage and the risk of development of an infection. Overall, coagulase negative staphylococci were cultured most often and in the pathogenic group *Enterobacterales*, were the most prevalent species.

In contrast with the postoperative situation, abdominal drains in place for malignant ascites seem to demonstrate different dynamics. In the study of Chan et al., drains were only colonized after a median period of 18 days, with staphylococci as the most prevalent bacteria [60]. Forty percent of patients with a colonized catheter in this patient group developed an infection.

Culturing abdominal drain fluid shortly after placement may be representative of the intra-abdominal infection for which the drain was inserted; however, after a certain period of time, external microorganisms may colonize the drain and subsequently enter the abdominal cavity or collection where the drain was placed. Reliable evidence about the interval between insertion and colonization is lacking. Studies on patients with indwelling urinary catheters show that after 24 h, 10% of urinary catheters are already colonized with ascending periurethral microbiota, and 18% of patients who are colonized with Gram-negative rods or enterococci develop bacteriuria, compared to 5% in non-colonized patients [61]. Extrapolating these observations, the culture results of abdominal drain fluid may only represent the true intra-abdominal situation in the first 12–24 h after placement. In all cases where abdominal drainage fluid is cultured, it is important to collect a fresh sample (i.e., after the replacement of the collection bag) and to take all precautions not to contaminate the sample. Tubings and tips are not suitable for routine procedures as they are not representative of the intra-abdominal situation [62]. Evidence from culturing drain tips or drain fluids in the early diagnosis of surgical site infections, in clean or clean contaminated surgery, found that while the negative predictive value is very high (99%), the positive predictive value is very low (11%) [63].

In all, the available clinical data make it difficult to draw any strong conclusions about the frequency of colonization of abdominal drains, the microorganisms involved, and the risk of subsequent infection. However, we can conclude that colonization is common, starts early in postoperative patients, and that the risk of colonization and/or infection increases over time.

## 5. Practical Approach

While it is highly likely that we will see a continued decrease in the use of abdominal drains in many types of elective surgery, drains remain an important element in the treatment of abdominal infections, in both surgical and non-surgical (i.e., percutaneous) management. In postoperative patients following surgery due to abdominal infection, drains may have a limited role for a short duration, but clear criteria for drain removal are absent.

From the above data and extrapolation from other settings, it is obvious that abdominal drains are prone to biofilm formation, especially in patients with abdominal infections. However, also in elective, clean, or clean-contaminated surgery, microbial biofilms are likely to develop, particularly if a drain remains in situ for a prolonged period of time.

While culturing abdominal drains is straightforward, differentiating between colonization and infection is challenging—if not impossible—from a microbiologist’s point of view. Overall, drains seem to be poor in predicting the presence of pathogens involved in any subsequent infection; on the other hand, drains may even play a role in maintaining infection or causing secondary or tertiary infection if they are used for prolonged periods of time.

**Figure 1:**
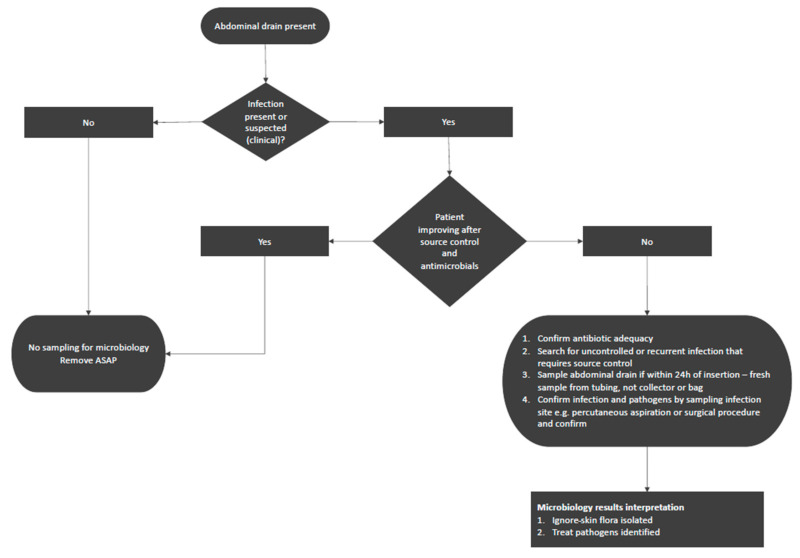
A practical approach to sampling abdominal drains in abdominal surgery patients.

Based on this, we propose a practical algorithm to assist decision making for hospitalized patients with abdominal drains (Figure 1). If no infection is suspected or if a patient is improving, obtaining and investigating routine abdominal fluid cultures from the drain is discouraged and removal of the drain as soon as possible is recommended. For patients who are not improving and in whom inappropriate therapy, a lack of source control, or other sources of infection have been excluded, sampling an abdominal drain may be of (limited) use. When interpreting the results of such cultures, it is important to ignore skin flora. It is preferred to obtain cultures from the infection site through percutaneous drainage or surgery to confirm the microbiological results and identify pathogens involved in the infectious process. Table 1 summarizes key abdominal drain management principles.

## 6. Conclusions

Abdominal drains are frequently used in hospitalized patients for both prophylactic and therapeutic indications, but the evidence for using drains in non-infectious surgery seems to be limited to a few specific situations only. When used, drains should be removed once the source has been controlled. Biofilm formation on abdominal drains is inevitable and may lead to the unnecessary use of antimicrobials if wrongly interpreted. Clinical data demonstrate that colonization is common and that it increases with the duration that a drain is left in situ. Samples obtained from such drains are not representative of pathogens involved in infections. We recommend limiting the use of drains and, when present, not to sample drains as a general rule.

## Figures and Tables

**Table 1:** Best practices related to abdominal drains.

Use of abdominal drains	Avoid the use of drains in non-pancreatic abdominal surgery
	Limit duration of abdominal drains in the treatment of abdominal sepsis
	Remove abdominal drains as soon as patient physiology allows
Sampling abdominal drains	Sample intra-operatively—NOT postoperatively—for reliable microbiology results
	Do not sample fluid from a drain that has been in situ for 24 h or longer
	Avoid culturing drains/parts of drains upon removal
Antimicrobial use	Ignore skin flora cultured from abdominal drains
	When infection is suspected clinically, do not solely target pathogens obtained from drain cultures

## Data Availability

Not applicable.
